# Early Weaning and Milk Substitutes Affect the Gut Microbiome, Metabolomics, and Antibody Profile in Goat Kids Suffering From Diarrhea

**DOI:** 10.3389/fmicb.2022.904475

**Published:** 2022-06-21

**Authors:** Tao Zhong, Cheng Wang, Xinlu Wang, Aline Freitas-de-Melo, Bo Zeng, Qianjun Zhao, Siyuan Zhan, Linjie Wang, Jiaxue Cao, Dinghui Dai, Jiazhong Guo, Li Li, Hongping Zhang, Lili Niu

**Affiliations:** ^1^Farm Animal Genetic Resources Exploration and Innovation Key Laboratory of Sichuan Province, College of Animal Science and Technology, Sichuan Agricultural University, Chengdu, China; ^2^Departamento de Biociencias Veterinarias, Facultad de Veterinaria, Universidad de la República, Montevideo, Uruguay; ^3^Institute of Animal Science, Chinese Academy of Agricultural Sciences (CAAS), Beijing, China

**Keywords:** caprine, CAZyme, immunoglobulins, serum biochemistry, serum metabolome, 16S rRNA, metagenomics

## Abstract

Early weaning and milk substitutes increase the incidence of diarrhea in young ruminants, which may modify their gut microbiota, metabolism, immunity, and health. The aim of the study was to determine if early weaning and milk substitutes affect the gut microbiota, metabolism, and immunological status of goat kids suffering from diarrhea. The 16S rRNA gene and metagenomic sequencing in feces and serum metabolomics of early-weaned and artificially reared goat kids suffering from diarrhea (DK group) and healthy goat kids reared by their mothers (HK group) were analyzed. The serum biochemistry and immunoglobulin concentration were also determined. Several probiotics, such as *Streptococcus* and *Lactobacillus*, were higher in the feces of the DK group than in feces of the HK group. *Ruminococcus* sp. was elevated in the feces of HKs, likely being a biomarker for goat health. Taking all the carbohydrate-active enzyme (CAZyme) families into consideration, 20 CAZyme families were different between the groups. Compared with the DK group, the relative quantity of glycoside hydrolases (GH) and glycosyltransferase (GT) families in the HK group decreased. GT70 was only identified in HK kids participating in the activity of β-glucuronosyltransferase during the carbohydrate metabolism. Overall, 24 metabolites were different between the groups, which were mainly involved in protein digestion and absorption, cyanoamino acid metabolism, and cholesterol metabolism. The concentrations of immunoglobulins G and M were significantly lower in the DK than in the HK group. In conclusion, our study characterized the fecal microbiota, metabolism, and immunological status of early-weaned and artificially reared goat kids suffering from diarrhea.

## Introduction

Early weaning is often performed in goat farms to accelerate the breeding cycle and increase milk production. However, young ruminants are prone to weaning stress and being separated from their mothers at an early age, and are often artificially reared with a milk substitute (Sevi et al., 2009). The stress caused by the early weaning (Orgeur et al., 1999; Freitas-de-Melo et al., 2022) and the difficulties in adapting to the milk substitute due to excessive dosage or inadequate temperature levels increased the number of youngs suffering from diarrhea (Grosskopf et al., 2017; Wang et al., 2019). Since the stress of early weaning negatively impacts the immunological status of youngs, it increases the susceptibility to diseases (Zhang et al., 2018), in particular infectious diarrhea (Skirnisson and Hansson, 2006; Fortuoso et al., 2019), caused by *Escherichia coli* (Wang W. U. et al., 2020), border disease virus (Wang W. U. et al., 2020), and parasitic diseases (Papanikolopoulou et al., 2018; Jacobson et al., 2020). Diarrhea is at fault for losses to farming enterprises, resulting in goat weight loss, growth retardation, and even death (Aldomy and Zeid, 2007). Therefore, it is necessary to generate potential biomarkers to support the development of practical tools for preventing and treating diarrhea in early-weaned and artificially reared goat kids.

In mammals, the gut microbiota is directly related to growth and development rates, nutrient digestion and absorption, immune response, and energy metabolism (Cummings and Macfarlane, 1997). Microorganisms in the gastrointestinal tract improve the host immune system and compete with harmful bacteria species for colonization sites, reducing the probability of bacterial infection in the gastrointestinal tract (Hooper et al., 2012). Before weaning, the microbial diversity and richness in the goat gut tend to increase with age, while the composition of the gut microbiome gradually matures in the early post-weaning period (at approximately 70 days of age) (Zhuang et al., 2020). In the first days after early weaning, bacterial diversity increases, and the composition of many dominant taxa is altered in the gut of lambs (Li et al., 2018). The artificial feeding provided after early weaning also triggers changes in the gut microbiota of goat kids, including the increases in the relative proportions of *Parasutterella*, *Megasphaera*, *Prevotellaceae*, *Akkermansia*, and *Subdoligranulum* (Wang et al., 2021). As mentioned before, artificial weaning followed by artificial rearing increases the frequency of diarrhea, which can also modify the gut microbiota. In fact, in goat kids suffering from diarrhea, *Bacteroides* remain the dominant species, and the proportion of *Clostridium* and *Paeniclostridium* increases, whereas that of *Rikenellaceae*, *Ruminococcaceae*, and *Christensenellaceae* decreases (Wang Y. J. et al., 2018). However, to the best of our knowledge, there is no information on how diarrhea induced by early weaning, followed by artificial rearing, changes the gut microbiota composition.

Early weaning and artificial rearing elicit metabolic and physiological changes, which impact both health and productive variables in ruminants, including the occurrence of diarrhea and loss of body weight (Orgeur et al., 1999). Furthermore, the plasma glucose concentration of early-weaned lambs was lower than that of suckling lambs (McCoard et al., 2020). Similarly, Suzuki et al. (2016) found that the ketone body level of weaned calves was lower than that of suckling calves, and the glucose metabolism of weaned calves had changed. Early weaning is a stressful situation for calves, which triggers an increase in the cortisol concentration (Prete et al., 2018) and also affects the immunological response of youngs, with a decrease being recorded in both globulin concentrations and antibody titer (Napolitano et al., 1995; Freitas-de-Melo et al., 2022). Hernández-Castellano et al. (2015) found that the lambs reared with their dams have higher serum immunoglobulin G (IgG) and IgM titers than those raised with a commercial milk substitute or cow milk during their early life. In addition, the diarrhea occurrence is positively related to the α2-globulin concentration in calves (Choi et al., 2021). Similarly, piglets suffering from infectious diarrhea have lower serum total protein, albumin, and globulin concentrations and digestive enzymes, such as disaccharidases, leucine aminopeptidase N, and alkaline phosphatase (Chethan et al., 2017). This study aims to determine if early weaning and milk substitutes affect the gut microbiota, metabolism, and immunological status of goat kids suffering from diarrhea.

## Materials and Methods

### Ethics Approval

All procedures involving lactating does and their kids were approved by the Institutional Animal Care and Use Committee at the College of Animal Science and Technology, Sichuan Agricultural University (China, SYXK2019-187). All animal experiments were based on the Regulations for the Administration of Affairs Concerning Experimental Animals (Ministry of Science and Technology, China, revised in June 2004).

### Animal Management, Experimental Design, and Sample Collection

The experiment was conducted at the National Conservation Farm of Chengdu Brown goat (Dayi County, Chengdu, China). The feeding and general management were performed following the guidelines for Chengdu Brown goat standards of Sichuan Province (DB51/T654-2007). Totally, 20 Chengdu Brown multiparous does carrying twin fetuses were randomly selected at 30 days of gestation. After delivery, all the goat kids were reared by their mothers indoor during the first 29 days of age. From then on, twins with similar body weights were assigned to two groups: (1) goat kids that remained being reared by their mothers (*n* = 20) and (2) goat kids that were weaned and artificially reared with milk substitutes (*n* = 20). The weaning was performed at 19:00 h. The artificially reared goat kids were fed twice daily with 120–200 mL at 0.2 g/mL of a milk substitute provided by artificial teats at 8:30 and 18:30 h, beginning at 30 days of age. The nutritional composition of milk replacer powder is shown in Supplementary Table 1. The artificially reared goat kids were housed in two pens (1 m^2^/kid), while the other group remained in the same paddock with their mothers. All goat kids were supplemented with solid concentrate starter [crude protein (17 g/kg), crude fiber (80 g/kg), crude ash (90 g/kg), Ca (10 g/kg), total phosphorus (4 g/kg), NaCl (4 g/kg), and lysine (4.5 g/kg)], forage (silage and hay), and water *ad libitum*.

**FIGURE 1:**
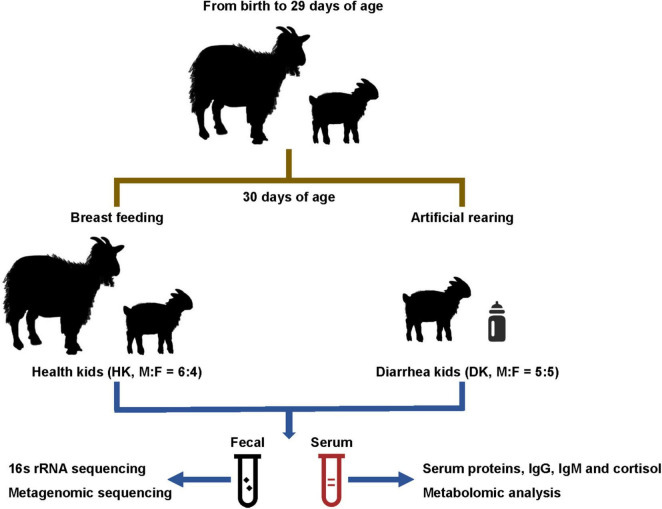
Experimental design and workflow.

At 30 or 31 days of age, fresh rectal fecal samples were collected simultaneously from 10 weaned kids that were observed having diarrhea (5 males and 5 females; DK group) and 10 healthy kids from the group that remained with their mothers (6 males and 4 females; HK group). Sterile cotton swabs were inserted into the rectum of each kid to collect fecal samples and were stored at −80°C. Fecal samples were collected twice from each individual at the same time. Thereafter, blood samples were collected by jugular venipuncture using vacuum tubes without anticoagulants, centrifuged at 3,000 r/min for 10 min to separate the serum, and then stored at −20°C. The fecal samples were collected for 16S rRNA and metagenomic sequencing, and the separated serum was used for biochemical, cortisol, and metabolomic determinations (Figure 1).

### DNA Extraction and Sequencing

Total microbial genomic DNA of feces was extracted using TGuide S96 Magnetic Stool DNA kits (Tiangen Biotech Co., Ltd., Beijing, China) in accordance with the manufacturer’s instructions. The purity and concentration of the isolated DNA were assessed by using a NanoDrop 1000 spectrophotometer (Thermo Fisher Scientific, Waltham, MA, United States). All the DNA samples were stored at −80°C until subsequent processing.

The universal bacterial primers (338F: 5′-ACTCCTACGG GAGGCAGCA-3′, 806R: 5′-GGACTACHVGGGTWTCTAAT-3′) were used to amplify the V3–V4 regions of the bacterial 16S rRNA gene. PCR amplicons were visualized by electrophoresis using a 2% agarose gel, purified with an E.Z.N.A.^®^ Cycle-pure kit (OMGEA, Doraville, GA, United States), and then quantified by using a Qubit 2.0 Fluorometer (Thermo Fisher Scientific, Waltham, MA, United States). After pooling, the amplicon libraries were sequenced on the Illumina Novaseq6000 platform (Illumina, San Diego, CA, United States).

The DNA sample was fragmented by using the ultrasonic interrupt method for metagenomics sequencing. Then, DNA fragments were purified and end-repaired, adding A to 3′ ends, and adding adaptors. Thereafter, agarose gel electrophoresis was used in fragment size selection, and PCR amplification was used to construct the sequencing library. Once the constructed libraries passed quality control, qualified libraries were sequenced on the Illumina PE150 sequencing platform.

### 16S rRNA Sequencing Data Analysis

Raw data were obtained through the Highseq platform, and the results were stored in the FASTQ (fq) file format. Usearch v10 (Edgar, 2017) software was used to cluster reads at a 97.0% similarity level. Using SILVA v138 (Glöckner et al., 2017) as a reference database, a naive Bayes classifier was used to make taxonomic annotations to the feature sequences. QIIME2 software (Bolyen et al., 2019) was used to obtain the species classification information corresponding to each feature, the composition of each sample community, and estimate the alpha diversity index and the beta diversity. The dominant bacterial community difference between groups was detected using the linear discriminant analysis (LDA) effect size (Segata et al., 2011).

### Metagenomic Analyses

Trimmomatic software (Bolger et al., 2014) was used to filter raw tags and get high-quality sequencing data. The clean reads data were obtained after aligning with the host genome sequence to remove the host contamination, and the sample sequencing data were quality-controlled. Then, the metagenome was assembled, and contig sequences shorter than 300 bp were filtered. MMseqs2 (Mirdita et al., 2019) software was used to remove redundancy, and the similarity threshold and coverage threshold were set to 95 and 90%, respectively. KEGG (Kyoto Encyclopedia of Genes and Genomes) database, eggNOG database, and GO (gene ontology) database were used. Hmmer (Wheeler and Eddy, 2013) software to find out all the families that meet the filtering threshold was used to compare the protein sequences of non-redundant genes with the hidden Markov model of each family in the carbohydrate-active enzyme (CAZyme) database.

### Metabolomic Analysis

The serum samples were thawed at 4°C, and 100 μL of the sample was mixed with 400 μL of cold methanol/acetonitrile (1:1, v/v) to remove the protein. The mixture was centrifuged for 15 min (14,000 *g*, 4°C). The supernatant was dried in a vacuum centrifuge. For the LC-MS analysis, a volume of 100 μL acetonitrile aqueous solution (acetonitrile:water = 1:1) was added for redissolution and then centrifuged for 15 min (14,000 *g*, 4°C). The supernatant was sampled and analyzed. The QC samples were prepared by pooling 10 μL of each sample and analyzed together with the other samples to monitor the stability and repeatability of instrument analysis. The QC samples were inserted regularly and analyzed within every five samples.

The samples were separated by an Agilent 1290 Infinity LC (Agilent, California, CA, United States) ultrahigh-performance liquid chromatography (UHPLC) HILIC column with the following parameters: column temperature 25°C, the flow rate 0.5 mL/min, injection volume 2 μL, and mobile phase composition A: 25 mM ammonium acetate and 25 mM ammonia hydroxide in water, B: acetonitrile. The gradient elution procedure was as follows: 0–0.5 min, 95% B; from 0.5 to 7 min, B changed linearly from 95 to 65%; 7–8 min, B changed linearly from 65 to 40%; 8–9 min, B remained at 40%; B changed linearly from 40 to 95% in 9–9.1 min, and remained at 95% in 9.1–12 min. The samples were placed in an automatic sampler at 4°C during the whole analysis. An AB Triple TOF 6600 mass spectrometer (AB SCIEX, Boston, MA, United States) was used to collect the first- and second-order spectrograms of samples. The electrospray source conditions for HILIC chromatography separation were as follows: ion source gas 1: 60, ion source gas 2: 60, curtain gas (CUR): 30, source temperature: 600°C, ion sapary voltage floating (ISVF) ± 5500 V (both modes); TOF MS scan m/z range: 60–1,000 Da, product ion scan m/z range: 25–1,000 Da, TOF MS scan accumulation time 0.20 s/spectra, and product ion scan accumulation time 0.05 s/spectra. The production scan was obtained by information-dependent acquisition (IDA) and a high-sensitivity mode. The parameters were set as follows: declustering potential (DP): 60 V (+) and −60 V (−); collision energy: 35 V with ± 15 eV; IDA: exclude isotopes within 4 Da; and candidate ions to monitor per cycle: 10.

The raw data in the Wiff format were converted to the MzXML format by ProteoWizard (Thermo Fisher Scientific, United States), and then XCMS software was used for peak alignment, retention time correction, and peak area extraction. Metabolite structure identification and data pretreatment were carried out for XCMS-extracted data, then the quality evaluation of experimental data was carried out. Compound identification of metabolites was performed by comparing accuracy m/z value (<25 ppm) and MS/MS spectra with an in-house database established with available authentic standards. All the raw data files are available at the Open Science Framework public data depository (MTBLS4035).

### Serum Biochemistry, Cortisol, and Immunoglobulin Concentrations

Total protein (TP), albumin (Alb), globulin (Glb), glucose (Glu), triglyceride (TG), blood urea nitrogen (BUN), total cholesterol (TCHO), immunoglobulin G (IgG), and immunoglobulin M (IgM) were analyzed by an automatic biochemical analyzer (Model 7020, Hitachi, Tokyo, Japan), utilizing commercial kits (Sichuan Maker Biotechnology Inc., Chengdu, China). Calibration controls were included for the measurements. Cortisol was determined using the goat ELISA kit (Meimian Industrial Co., Ltd., Nanjing, China) following the manufacturer’s instructions. The sensitivity of the measurement was 1.0 ng/mL, and the intra assay coefficient of variation was 10%.

### Statistical Analysis

Statistical analysis was performed by SPSS 27 (Chicago, IL, United States). All data were tested for normal distribution using the Shapiro–Wilk test. The Wilcoxon rank-sum test was used to compare the means of two groups. A significant change was observed with a linear discriminant analysis (LDA) score > 3.5 calculated by LEfSe. Spearman’s rank correlations were performed between the 24 different metabolites and gut microbes. A level of *P*-value < 0.05 was considered statistically significant.

## Results

### Diarrhea Induced a Shift in the Gut Microbiota Composition of Early-Weaned and Artificially Reared Goat Kids

**FIGURE 2:**
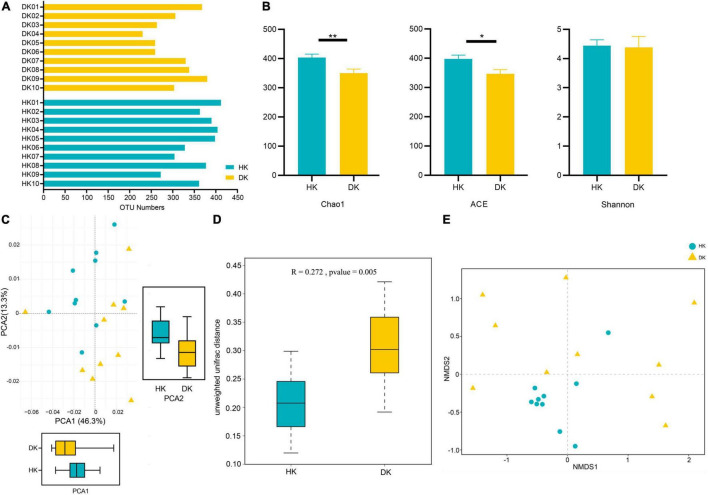
Comparison of fecal microbial community structures between diarrheic and healthy goat kids. **(A)** OTU numbers in each sample. **(B)** Alpha diversity analysis, and data represent mean values ± standard error of mean (SEM) *n* = 10. **(C)** Principal component analysis (PCA), and sample distance indicates the similarity of composition between the two samples. **(D)** ANOSIM based on unweighted UniFrac for differences, and the *R* value indicates the group difference, *n* = 10. **(E)** NMDS analysis based on unweighted UniFrac, and the distance between points indicates the degree of sample variation. *Means *P* < 0.05, **means *P* < 0.01.

The V3 + V4 hypervariable regions of the 16S rRNA gene were sequenced to identify the fecal bacterial community diversity and composition in diarrheic and healthy goat kids (Figure 1). A total of 3,200,413 sequences were acquired from 10 healthy goat kids (HK) and 10 goat kids suffering from diarrhea (DK) (Supplementary Table 2). Totally, 507 operational taxonomic units (OTUs) based on 97% similarity were detected, with an average of 360 OTUs in the HK group and 303 OTUs in the DK group (Figure 2A). The alpha diversity analysis showed that the gut microbial Chao1 index (*P* = 0.0085) and ACE index (*P* = 0.0168) of the DK group were significantly lower than those of the HK group. The Shannon index was not different between the groups (Figure 2B). The beta diversity was assessed by the principal component analysis (PCA), and the results showed differences in the composition of intestinal microbiota between the groups (Figure 2C). The analysis of similarities (ANOSIM) based on unweighted UniFrac for differences between groups was also significant (*R* = 0.272, *P* = 0.005, Figure 2D). The non-metric multi-dimensional scaling (NMDS) plot displaying the dissimilarity of the microbial community also had a structural distinction between the groups (Figure 2E).

**FIGURE 3:**
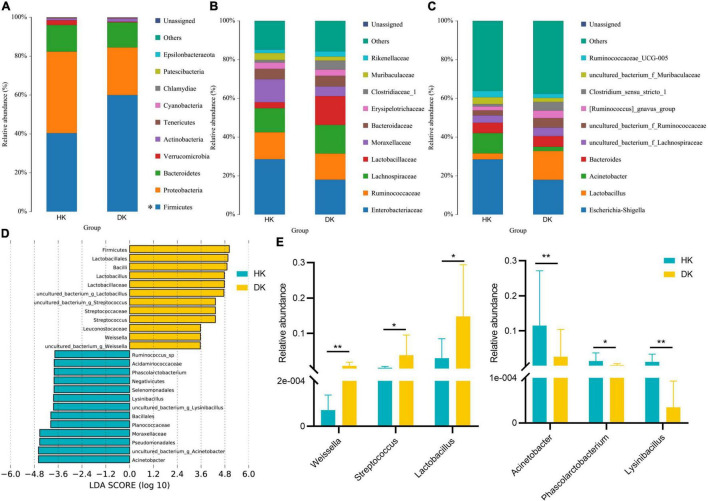
Taxonomic profiles of the fecal bacteria in diarrheic and healthy goat kids from 16S rRNA gene sequencing. **(A)** Relative abundance of the top 10 phylum. **(B)** Relative abundance of the top 10 families. **(C)** Relative abundance of the top 10 genus. A color represents a species, and the block length indicates the relative abundance of the species. Other species are grouped into others as shown in the figure, with unclassified representing species that have not been taxonomic annotated, *n* = 10. **(D)** LEfSe bar represents abundant differential taxa in goat kids’ fecal microbiome. The length of the bar chart represents the impact of different species. **(E)** Different genera in diarrheic and healthy goat kids (*P* < 0.05), and data represent mean values ± standard error of mean (SEM), *n* = 10. * Means *P* < 0.05, ** means *P* < 0.01.

Differences in the major taxonomical fecal microbiota profiles between the DK and HK groups were further identified (Figures 3A–C). The most relatively abundant phylum in the two groups was the same, Firmicutes and Proteobacteria. The relative abundance of Firmicutes in the DK group was significantly higher than in the HK group (*P* = 0.01, Figure 3A). The ratio of Firmicutes to Bacteroidetes was different between the groups (F/B = 2.7893 in HK and F/B = 4.4847 in DK). At the family level, *Enterobacteriaceae* was the most abundant in the HK and DK groups, followed by *Ruminococcaceae* and *Lachnospiraceae* (Figure 3B). At the genus level, *Escherichia and Shigella* were the dominant bacteria in both groups. Moreover, the dominant bacteria were different between the groups. *Lactobacillus* was the dominant bacteria in the DK group (*P* = 0.02, Figure 3E), and *Acinetobacter* was the dominant bacteria in the HK group (*P* = 0.008, Figure 3E).

There were significant differences in the major taxonomical profiles (mean relative abundance > 0.1%) of fecal microbiota between the groups (Supplementary Table 3). The linear discriminant analysis effect size (LEfSe) showed that the diversity of gut microflora was dramatically altered by diarrhea. *Weissella*, *Streptococcus*, and *Lactobacillus* played a crucial role in the DK group at the genus level (Figure 3D). The relative abundance of *Arcobacter*, *Phascolarctobacterium*, and *Lysinibacillus* decreased in the DK group (Figure 3E). In general, the structure of fecal microflora of the DK group was different from that of the HK group.

### Metagenome-Wide Association of Microbiome Features

**FIGURE 4:**
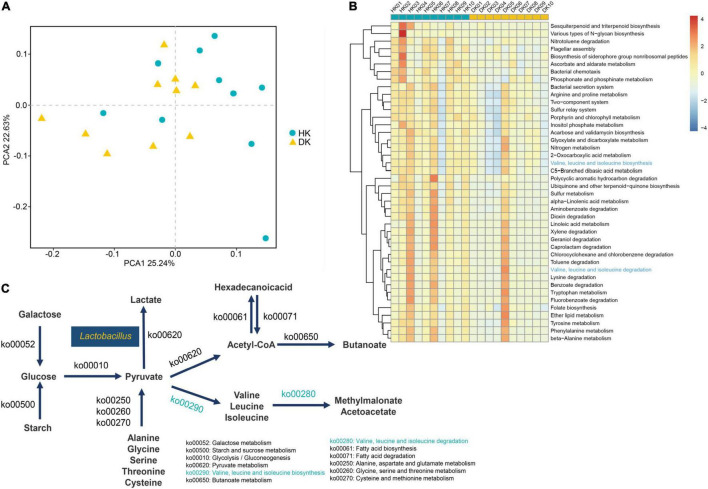
**(A)** PCoA used binary Jaccard distances, and the distance on the coordinate diagram indicates the similarity of functional composition between samples. **(B)** Hierarchical clustering of functional genes whose *P*-values < 0.05 after the rank-sum test. The different KEGG pathways in level 3. **(C)** Microbial functions and species involved in carbohydrate metabolism and amino acid metabolism in fecal of goat kids, and yellow means increased in the DK group, and blue means increased in the HK group.

Metagenomic sequencing was used to determine the microbial gene functions in the HK and DK groups. After trimming the low-quality reads, host sequences, assembling, gene prediction, and construction, a total of 3,419,770 non-redundant genes were identified (Supplementary Table 4). Through gene annotation by aligning sequences to the Kyoto Encyclopedia of Genes and Genomes (KEGG) database, a total of 1,048,576 KEGG genes were identified. Then, the binary Jaccard distances of each pair of samples were calculated, and differences between the groups were identified (Figure 4A). To figure out the specific functionalities contributed to the bacterial differences between the groups, significance tests of distances between the HK and DK groups were performed in all KEGG pathways level 3 (Supplementary Table 5). There were 42 different pathways in the pathways level 3 (Figure 4B, *P* < 0.05); amino acid metabolism [such as valine, leucine, and isoleucine biosynthesis (ko00290); valine, leucine, and isoleucine degradation (ko00280); and tryptophan metabolism (ko00380)] which had increased relative abundance in the HK group (Figure 4C).

**FIGURE 5:**
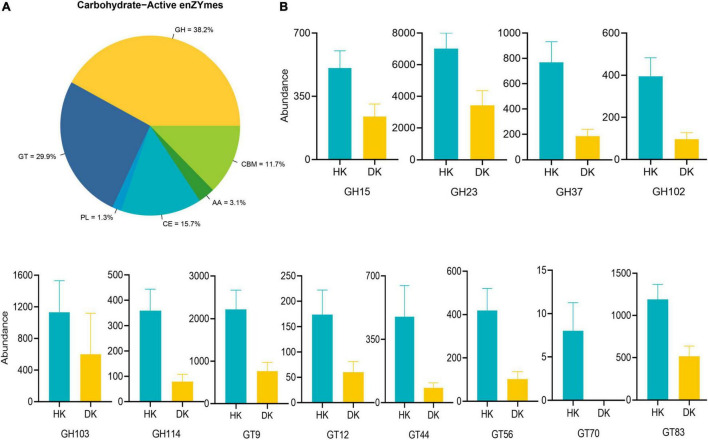
Comparisons of the total abundance of CAZyme genes of fecal microbiomes of goat kids. **(A)** Carbohydrate enzyme distribution scale diagram. Colors indicate functional categories. **(B)** Comparisons of the relative abundance of the CAZyme gene families of the fecal microbiomes of goat kids in the diarrheic and healthy goat kids, and data represent mean values ± standard error of mean (SEM) (*P* < 0.05, *n* = 10).

A screen for CAZymes in the assembled contigs was performed to explore the microbial potential for dietary degradation in each experimental group. The DK group showed a lower abundance of CAZymes than the HK group, and these relative genes were assigned to 333 different families, including 11 families of auxiliary activities (AAs), 77 families of carbohydrate-binding modules (CBMs), 140 families of glycoside hydrolases (GHs), 83 families of glycosyltransferases (GTs), 16 families of carbohydrate esterases (CEs), and 22 families of polysaccharide lyases (PLs, Supplementary Table 6 and Figure 5A). To further determine the enzymes that play a crucial role in carbohydrate degradation, a genetic difference analysis was performed. Taking all the CAZyme families into consideration, 20 CAZyme families were different between groups (*P* < 0.05, Supplementary Table 7). The quantity of these different enzymes was higher in the HK than in the DK group. Most of them come from GTs and GHs (Figure 5B), and interestingly, GT70 was found only in the HK group. In addition, to understand the role of these enzymes in carbohydrate metabolism, the activity of these differential families was explored. These families of enzymes participated in different processes of sugar metabolism. α-Glucosyltransferase was classified as the GT44 family, glucoamylase and glucodextranase were classified as the GH15 family, β-glucuronosyltransferase was classified as the GT70 family, and glucooligosaccharide oxidase was classified as the AA7 family (Supplementary Table 7).

### Serum Metabolome Identified a Microbiome That Functioned *via* Lactate Metabolism

Untargeted metabolomics analyses were performed to identify the serum metabolic profiles in the HK and DK groups. PCA was performed on the peaks extracted from all experimental and quality control (QC) samples in positive (Supplementary Figure 1A) and negative ion modes (Supplementary Figure 1B). The QC samples in the positive and negative ion modes were closely clustered together, indicating good repeatability of the experiment. In this study, 327 metabolites were identified by the combination of positive and negative ion models, of which 187 and 140 metabolites were identified by positive and negative ion models, respectively (Supplementary Table 8). We found that among all the metabolites, except the undefined metabolites, organic acids and derivatives (70, 21.407%) and lipids and lipid-like molecules (48, 14.679%) were the two most abundant metabolites (Supplementary Figure 1C).

**FIGURE 6:**
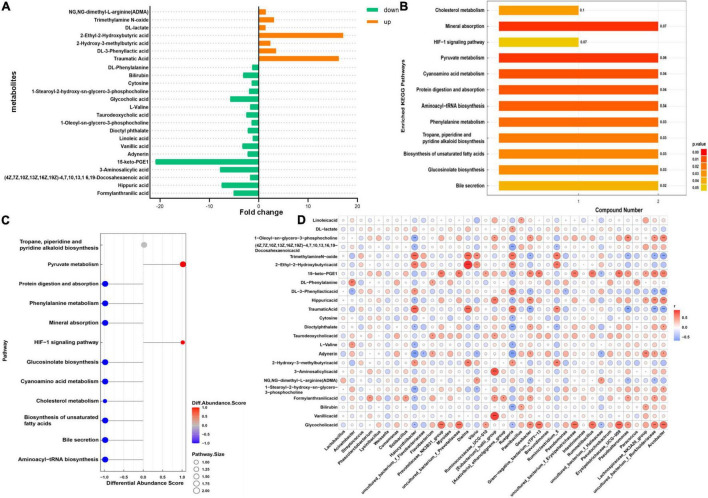
Gut metabolome of diarrheic and healthy goat kids. **(A)** Different metabolites in the two groups, and fold change represents differential expression multiple. **(B)** KEGG enrichment pathway diagram, the vertical axis represents each KEGG metabolic pathway, and the horizontal axis represents the number of differentially expressed metabolites contained in each KEGG metabolic pathway. Color represents the *P*-value of enrichment analysis. **(C)** Differential abundance score plot of differential metabolic pathways. The DA score is the overall total change of all metabolites in the metabolic pathway. **(D)** Heatmap of correlations between differential metabolites and bacterial genus (Spearman’s rank correlations). The darker the color, the greater the absolute value of r. * Means 0.01 < *P* < 0.05, ** means 0.001 < *P* < 0.01, and *** means *P* < 0.001.

VIP > 1 and *P* < 0.05 were set as the standard for screening differential metabolites with a significant difference, as shown in Figure 5B; 24 metabolites were found in the positive and negative ion models (Figure 6A). Subsequently, KEGG analysis for all differential metabolites showed that these differential metabolites were significantly enriched in 12 metabolic pathways (*P* < 0.05), and there were 1–2 different metabolites in each metabolic pathway (Figure 6B). The differential abundance scores for each pathway were calculated to explore the overall changes of metabolites in these pathways. In DK goat kids, the majority of metabolic pathways decreased, and only two pathways increased (Figure 6C). Interestingly, most of the decreased pathways in the DK group were involved in protein digestion and absorption, mineral absorption, cyanoamino acid metabolism, and cholesterol metabolism (Figure 6C). To facilitate the observation of the expression of differential metabolites in the KEGG metabolic pathway, the KEGG metabolic pathways with greater than or equal to five differential metabolites were selected for cluster analysis. Bilirubin, DL-lactate, DL-phenylalanine, and L-valine were involved in metabolic pathways (ko01100, Supplementary Figure 2A) and the biosynthesis of secondary metabolites (Supplementary Figure 2B). Overall, 12 metabolites and 11 bacterial genera were strong correlated (| r | > 0.6, *P* < 0.05, Figure 6D).

### Serum Biochemistry and Cortisol and Immunoglobulin Concentrations

**FIGURE 7:**
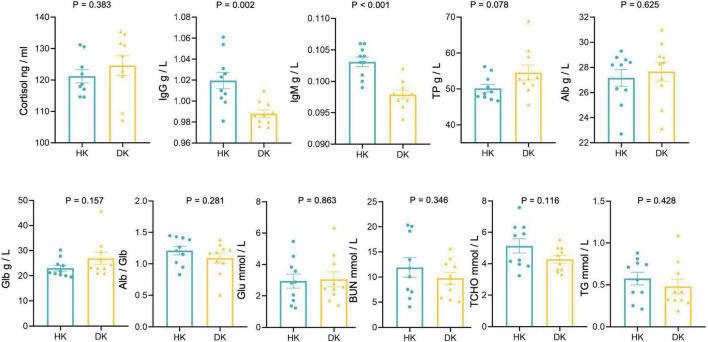
Serum biochemical and cortisol measurements of diarrheic and healthy goat kids; data represent mean values ± standard error of mean (SEM), *n* = 10.

Cortisol concentration did not differ between the groups (Figure 7). IgG (*P* = 0.002) and IgM (*P* < 0.001) concentrations were lower in the DK group than in the HK group. However, serum TP, Alb, Glb, Glu, TG, BUN, TCHO, IgG, IgM, and Alb/Glb concentrations did not differ between the groups (Figure 7).

## Discussion

### Fecal Microbiome Composition Impacted Host Health Status

We described the fecal microbiome changes in the structure and function for the first time in early-weaned and artificially reared goat kids suffering from diarrhea. We found differences between DK and HK goat kids in the relative microbial abundance at different levels. The HK group had more OTUs than the DK group, and the richness index (Chao1 and ACE) of the HK group was significantly higher than that of the DK group. In general, higher microbiota richness and diversity are considered both beneficial and protective for the host (Rinninella et al., 2019). These results are also consistent with those of Yang et al.’s (2019) study on piglets, suggesting that diarrhea can change the composition of intestinal microflora in goat kids.

Many studies have reported that the phylum Firmicutes and Bacteroidetes dominate the fecal microbiota of piglets (Looft et al., 2014), cattle (Alipour et al., 2018), goats (Wang Y. J. et al., 2018), and sheep (Belanche et al., 2019), consistent with our findings. An elevated Firmicutes-to-Bacteroidetes ratio has been associated with different diseases (Ley et al., 2006; Rajilić-Stojanović et al., 2011). We found that the relative abundance of Firmicutes in the DK group was higher than that in the HK group (Figure 3A). Changes in the relative abundance of Firmicutes in the intestinal tract of DK goat kids modified the ratio of Firmicutes to Bacteroidetes (F/B = 2.7893 in HK and F/B = 4.4847 in DK), which likely negatively affected the immunity of DK goat kids. Therefore, we speculated that diarrhea in early-weaned and artificially reared goat kids made them even more susceptible to infections. Complementarily, although the relative abundance of *Lactobacillus* decreases in neonatal piglets suffering from diarrhea (Yang et al., 2017), we found that the relative abundance of *Lactobacillus* in the intestinal tract of the DK group was higher than that in the HK group (Figure 3E). *Lactobacillus* likely avoided the worst immunity and health status in the DK group as it prevents pathogens from overgrowing in the intestine (Charlet et al., 2020). On the other hand, *Ruminococcus* sp. elevated in the feces of HK, likely being a biomarker for goat health (Figure 3D). As was revealed in post-weaned piglets, the abundance of *Ruminococcus* in diarrheic piglets is lower than that in healthy ones (Yang et al., 2019). *Ruminococcus* is a critical participant in nutrient metabolism, including carbohydrate fermentation and polysaccharide and steroid metabolism, and is crucial for maintaining the normal physiological function of the intestine (Hess et al., 2011). Therefore, we suggest that the decreased abundance of *Ruminococcus_*sp reduced the ability of intestinal epithelial cells to digest and absorb carbohydrates and perhaps even induced diarrhea in goat kids.

### Microbiome of Healthy Goat Kids Had Better Carbohydrate Digestion and Absorption Capacity

CAZymes are several enzymes that assemble or break down oligosaccharides and polysaccharides. The presence of many CAZyme genes in the gut bacterial genome is quite likely the result of gene replication and/or horizontal gene transfer among different bacteria (El Kaoutari et al., 2013). Studies have shown that GHs and PLs are two critical CAZymes for the degradation of different substrates (Lombard et al., 2010). In this study, we found that enzymes with differences between the groups in relative abundance mainly came from GHs and GTs, while enzymes with no significant differences between the groups came from PLs. Accordingly, we paid more attention to the changes in GHs, and it was noteworthy that the relative abundance of GHs in the HK group was higher than that in the DK group (Figure 5B). Among these GHs, GH15 otherwise known as amyloglucosidase, mainly came from *Arthrobacter* and *Corallococcus* (Li et al., 2017). Remarkably, these bacteria were present in the feces of both groups of goat kids, suggesting that these genera are involved in the decomposition of complex polysaccharides in the intestinal tract of goat kids. GH114 members are present in *Streptomyces* (Olanrewaju and Babalola, 2019), and the relative abundance of *Streptomyces* in the HK group was greater than that in the DK group (Supplementary Figure 3). GH23 derived from Firmicutes is fundamental for starch decomposing (Srivastava et al., 2020). Moreover, different feeding patterns in sheep can cause changes in the expression of genes, encoding the GH family in the rumen (Lin et al., 2019). These results support the hypothesis that early weaning, followed by a dietary change, modifies the expression of enzymes in the host gastrointestinal tract, affecting the digestive process and causing diarrhea in goat kids.

### The Metabolic Capacity of Goat Kids Differed Between Those Goats Suffering From Diarrhea and Healthy Goat Kids

We found a total of 24 differential metabolites in the serum of HK and DK groups, among which DL-lactate attracted our attention. Lactate has been shown to inhibit the growth of some pathogenic bacteria, including *Escherichia coli* (McWilliam Leitch and Stewart, 2002). However, it may also promote the development of specific pathogens, such as *Salmonella* (Wang S. P. et al., 2020), which causes diarrhea in animals and inhibits the production of butyrate (Gillis et al., 2018). In our study, the DL-lactate level in the serum of the DK group was higher than that in the HK group (Figure 6A), which may be caused by the increased abundance of *Lactobacillus* in feces of the DK group (Figure 3E). The higher concentration of serum lactate in the DK group could also be caused by the stress response at weaning, as weaned youngs increase their locomotion (Menant et al., 2022) and, consequently, the concentration of lactate in the blood (Çakmakçi et al., 2021).

In addition, DL-phenylalanine and L-valine were enriched in the cyanoamino acid metabolism pathway. Amino acids have been shown to play a role in the function of the immune system (Kelly and Pearce, 2020), while in the present study, DL-phenylalanine was downregulated in the serum of DK goat kids (Figure 6). Lee et al. (2020) found that phenylalanine is one of the potential biomarkers distinguishing patients with irritable bowel syndrome from healthy ones. L-valine is important for animal health and metabolism and is produced by microbial fermentation (Wang X. Y. et al., 2018). It can upregulate pro-inflammatory cytokines and downregulate anti-inflammatory cytokines in the immune system (Zhang et al., 2017). The L-valine detected in the serum of the DK group was significantly lower than that in the HK group (Figure 6A), so we speculated that the difference between the groups in gut microbial fermentation might be a consequence of this, which further affected the health of goat kids.

### The Antibody Profile Differed Between Goat Kids Suffering From Diarrhea and Those That Are Not

The IgG and IgM concentrations, the primary serum immune markers, were significantly lower in the DK than in the HK group. Although cortisol commonly is one of the causes for a worsening immunological status of animals, this was not the case as we found no difference between groups. Furman-Fratczak et al. (2011) found that serum IgG concentration was positively associated with calf health. We proposed that the decrease in IgG and IgM in DK could be caused by weaning as it decreases the antibody titer in lambs (Napolitano et al., 1995). Diarrhea also could decrease circulating immunoglobulins lost through epithelial exudation in the area of gut mucosal damage (Varricchi et al., 2021). Moreover, the greater relative abundance of Firmicutes and Firmicutes-to-Bacteroidetes ratio was identified in the DK group, and both were negatively correlated with IgG and IgM levels (Shen et al., 2018).

Serum biochemistry can partly reflect the metabolism and the health status of animals. Serum TP, Alb, and BUN reflect the metabolic rate of protein in the body to a certain extent. Even though the metabolites found in the serum of the DK group were mainly involved in protein digestion and absorption and cholesterol metabolism, we found no differences between the groups in serum TP, Alb, BUN, and TCHO concentrations. On the other hand, the gut microbiota of the HK goat kids had a better ability to digest and absorb carbohydrates; however, we did not find a greater serum glucose concentration in that group as we expected. Despite the fact that serum globulin was closely related to the immune level of the body (Geng et al., 2021) and decreased after weaning in sheep (Freitas-de-Melo et al., 2022), we did not find differences between the groups in globulin concentrations.

In summary, our study characterized the gut microbiota, metabolism, and immunological status of early-weaned and artificially reared goat kids suffering from diarrhea. The diversity and richness of bacteria in the fecal of HK group led to a higher abundance of enzymes involved in carbohydrate metabolism. Due to the changes in the microbial structure and function, there were differences in nutrient absorption, transportation, and metabolism between the groups. The metabolites found in the serum of the DK group were mainly involved in protein digestion and absorption, and cholesterol metabolism. The abundance of *Lactobacillus* in the gut of the DK group was higher than that in the HK group, which likely prevented pathogens from overgrowing in the intestine of DK kids. *Ruminococcus* sp. was elevated in the feces of the HK group, likely being a biomarker for goat health. The antibody profile of the HK group was better than that of the DK group, and thus, the latter goat kids had a worse immunological status than the HK group. Overall, the microbiome changes in early-weaned and artificially reared goat kids suffering from diarrhea affected the metabolic process of nutrients in the host. Our study provides insights into support of the development of practical tools for preventing and treating diarrhea in early-weaned and artificially reared goat kids.

## Data Availability Statement

The datasets presented in this study can be found in online repositories. The names of the repository/repositories and accession number(s) can be found below: National Center for Biotechnology Information (NCBI) BioProject, https://www.ncbi.nlm.nih.gov/bioproject/, PRJNA792435 and The European Molecular Biology Laboratory’s European Bioinformatics Institute (EMBL-EBI) MetaboLights, MTBLS4035.

## Ethics Statement

The animal study was reviewed and approved by the Institutional Animal Care and Use Committee at the College of Animal Science and Technology, Sichuan Agricultural University.

## Author Contributions

TZ and LN conceived and designed the experiments. CW and XW collected the samples and executed the experiments. CW and TZ analyzed the data and wrote the original draft. BZ, QZ, SZ, LW, JC, DD, JG, LL, and HZ participated in methodology. AF-d-M contributed to the interpretation and made substantial contributions to the manuscript. All authors have read and approved the final manuscript.

## Conflict of Interest

The authors declare that the research was conducted in the absence of any commercial or financial relationships that could be construed as a potential conflict of interest.

## Publisher’s Note

All claims expressed in this article are solely those of the authors and do not necessarily represent those of their affiliated organizations, or those of the publisher, the editors and the reviewers. Any product that may be evaluated in this article, or claim that may be made by its manufacturer, is not guaranteed or endorsed by the publisher.

## Supplementary Material

Supplementary Figure 1 **(A)** PCA of population samples with positive and negative ion patterns. **(B)** The proportion of metabolites in each chemical classification. **(C)** Carbohydrate enzyme distribution scale diagram.

Supplementary Figure 2 Clustering heat map of differential metabolites in KEGG pathway. **(A)** ko01100: Metabolic pathways. **(B)** ko01110: Biosynthesis of secondary metabolites.

Supplementary Figure 3 Streptomyces relative abundance map with metagenomic species annotation results.
