# Supplementary figures and images for: Early Weaning and Milk Substitutes Affect the Gut Microbiome, Metabolomics, and Antibody Profile in Goat Kids Suffering From Diarrhea
Source: Front Microbiol. 2022 Jun 21;13:904475. doi: 10.3389/fmicb.2022.904475 (PMC9253616; doi:10.3389/fmicb.2022.904475)

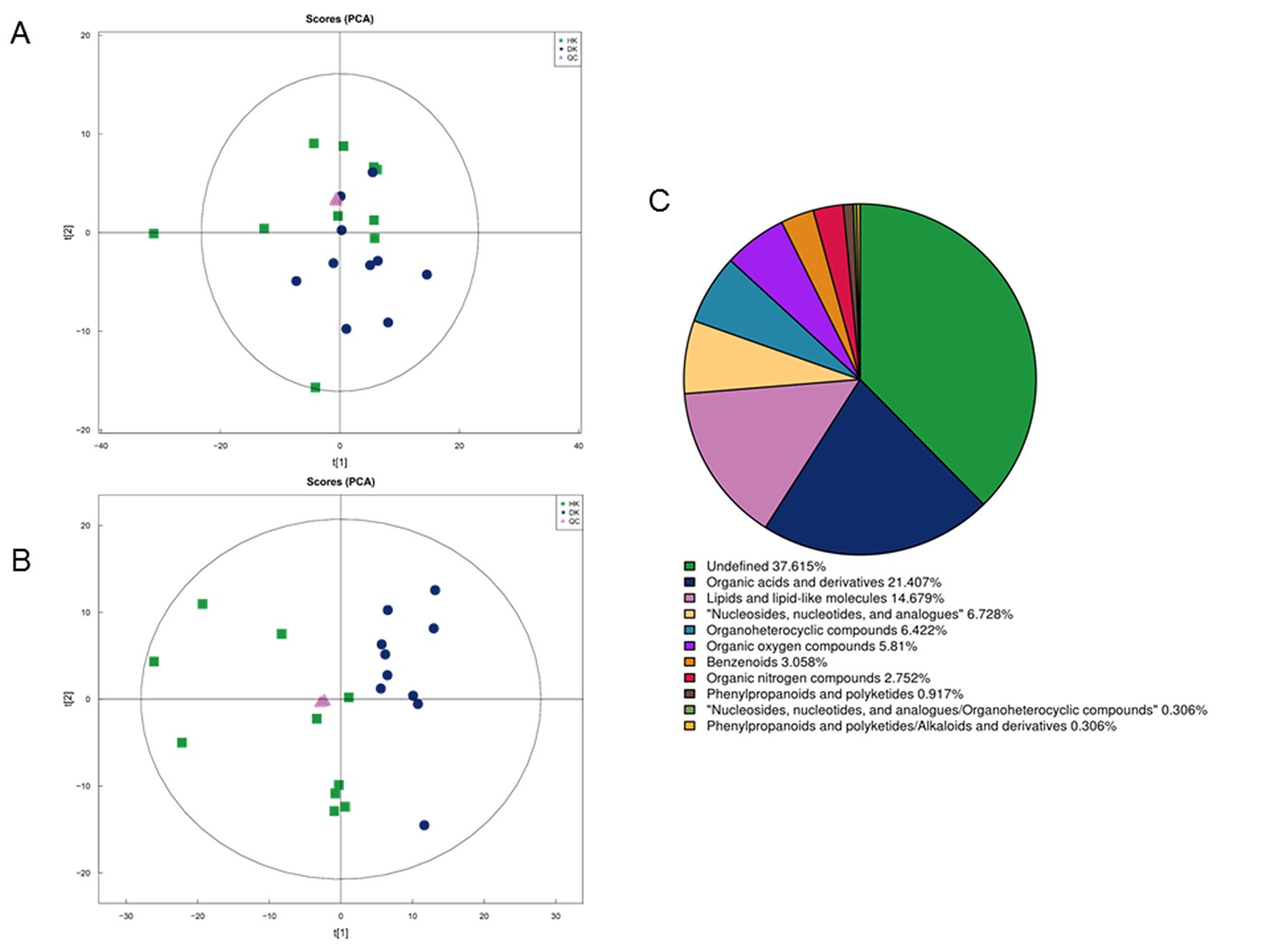

Supplement: Supplementary Figure 1 — (A) PCA of population samples with positive and negative ion patterns. (B) The proportion of metabolites in each chemical classification. (C) Carbohydrate enzyme distribution scale diagram. [file Image_1.TIF]

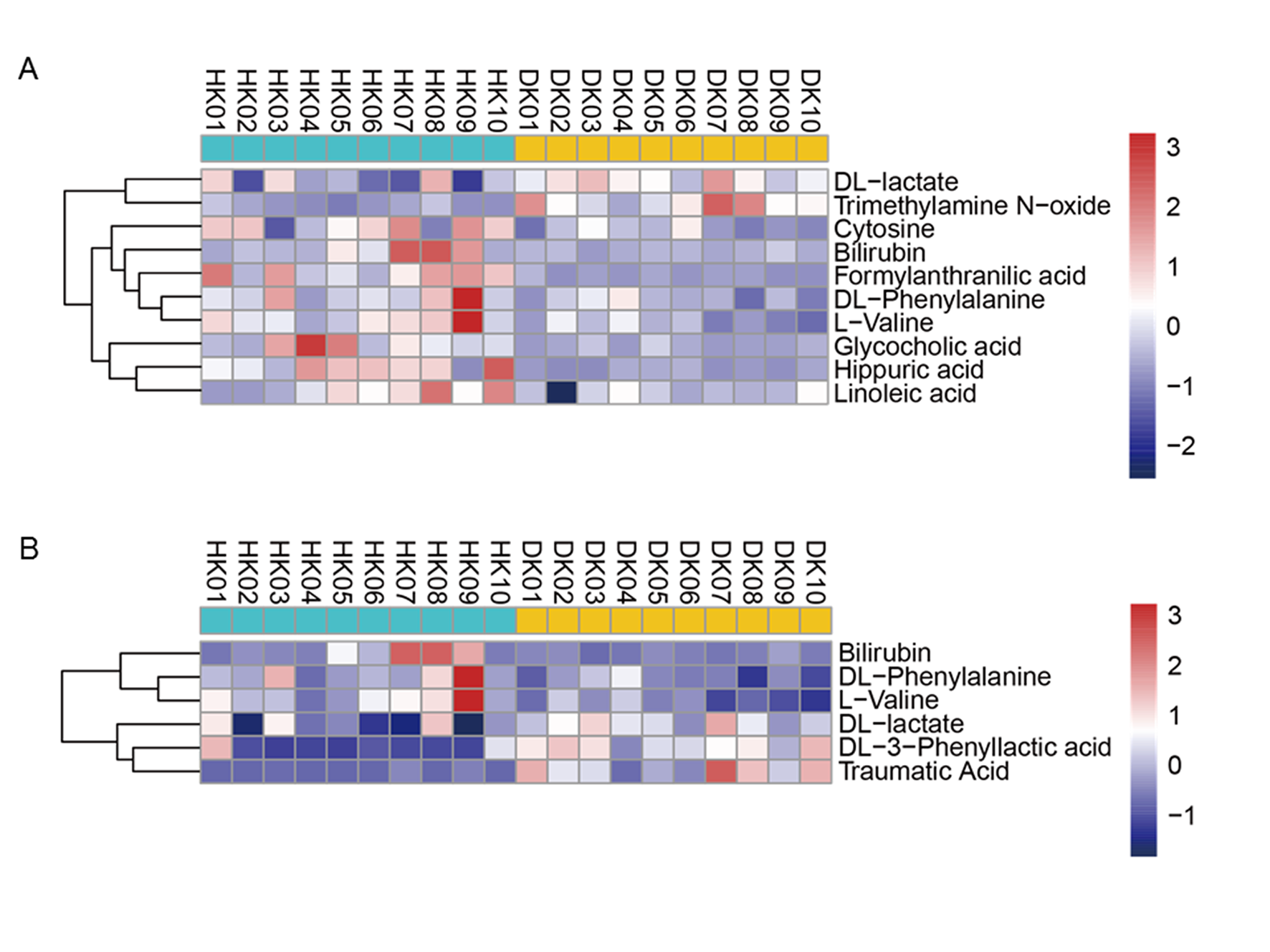

Supplement: Supplementary Figure 2 — Clustering heat map of differential metabolites in KEGG pathway. (A) ko01100: Metabolic pathways. (B) ko01110: Biosynthesis of secondary metabolites. [file Image_2.TIF]

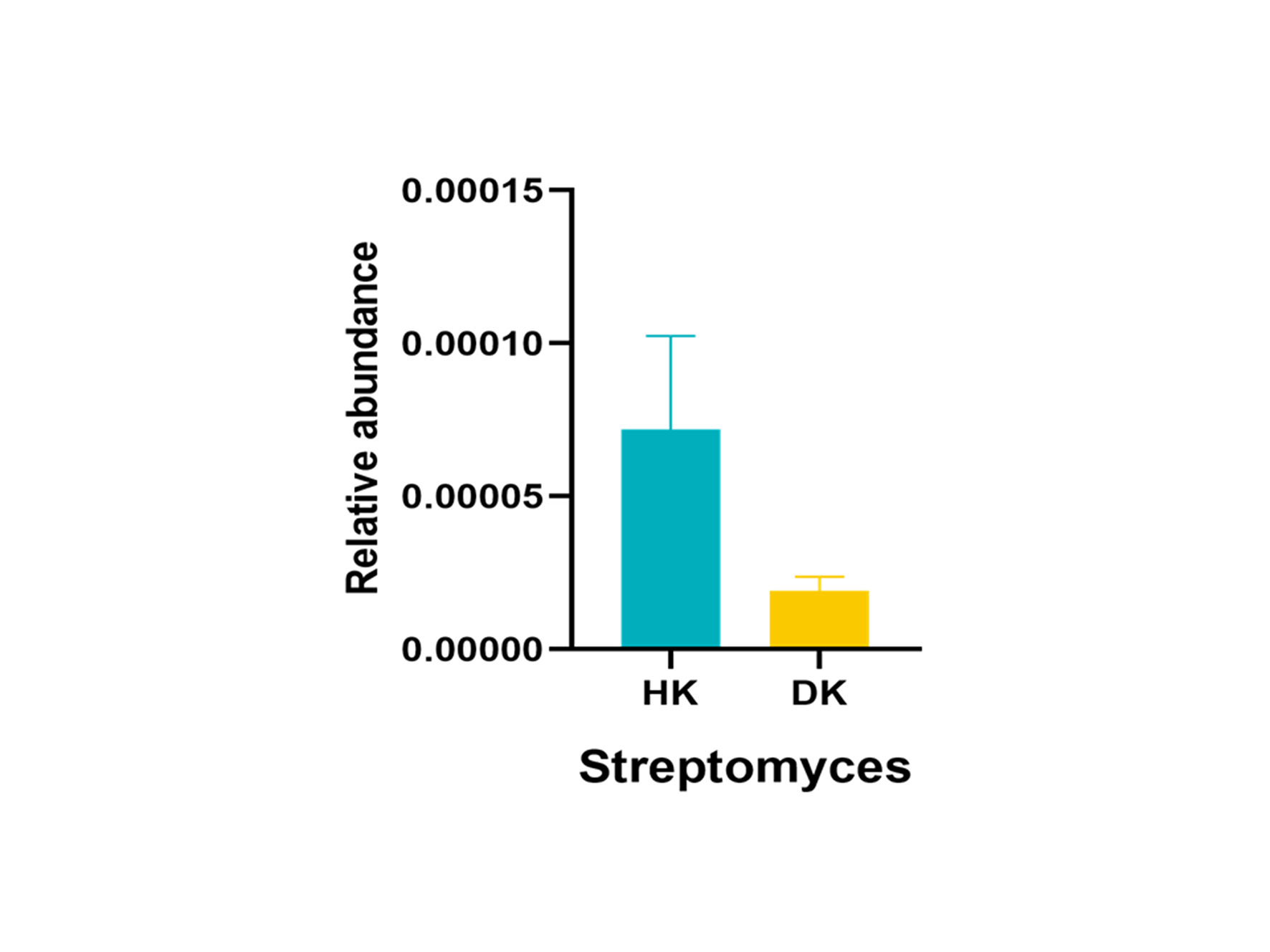

Supplement: Supplementary Figure 3 — Streptomyces relative abundance map with metagenomic species annotation results. [file Image_3.TIF]
